# The role of ^18^F−FDG PET in predicting the pathological response and prognosis to unresectable HCC patients treated with lenvatinib and PD-1 inhibitors as a conversion therapy

**DOI:** 10.3389/fimmu.2023.1151967

**Published:** 2023-05-05

**Authors:** Guanyun Wang, Wenwen Zhang, Xiaohui Luan, Zhanbo Wang, Jiajin Liu, Xiaodan Xu, Jinming Zhang, Baixuan Xu, Shichun Lu, Ruimin Wang, Guangyu Ma

**Affiliations:** ^1^ Department of Nuclear Medicine, The First Medical Centre, Chinese People's Liberation Army (PLA) General Hospital, Beijing, China; ^2^ Nuclear Medicine Department, Beijing Friendship Hospital, Capital Medical University, Beijing, China; ^3^ Faculty of Hepato-Pancreato-Biliary Surgery, Chinese People's Liberation Army (PLA) General Hospital/Institute of Hepatobiliary Surgery of Chinese People's Liberation Army/Key Laboratory of Digital Hepetobiliary Surgery, People's Liberation Army, Beijing, China; ^4^ Graduate School, Medical School of Chinese People's Liberation Army (PLA), Beijing, China; ^5^ Department of Pathology, The First Medical Centre, Chinese People's Liberation Army (PLA) General Hospital, Beijing, China

**Keywords:** unresectable hepatocellular carcinoma, conversion therapy, major pathological response, prognosis, ^18^F-FDG PET

## Abstract

**Purpose:**

To investigate the diagnostic value of ^18^F-fluorodeoxyglucose positron emission tomography (^18^F-FDG PET), as an imaging biomarker, for predicting pathological response and prognosis of unresectable hepatocellular carcinoma (HCC) patients treated with Lenvatinib and programmed cell death protein 1 (PD-1) inhibitors as a conversion therapy.

**Methods:**

A total of 28 unresectable HCC patients with BCLC stage B or C were treated with Lenvatinib and PD-1 inhibitors before surgery. The ^18^F-FDG PET/CT scans were acquired before pre- (scan-1) and post-conversion therapy (scan-2). The maximum standardized uptake value (SUVmax), TLR (tumor-to-normal liver standardized uptake value ratio), and the percentages of post-treatment changes in metabolic parameters (ΔSUVmax [%] and ΔTLR [%]) were calculated. Major pathological response (MPR) was identified based on the residual viable tumor in the resected primary tumor specimen (≤10%). Differences in the progression-free survival (PFS) and overall survival (OS) stratified by ΔTLR were examined by the Kaplan-Meier method.

**Results:**

11 (11/28, 39.3%) patients were considered as MPR responders and 17 (17/28, 60.7%) patients as non-MPR responders after conversion therapy. ΔSUVmax (-70.0 [-78.8, -48.8] vs. -21.7 [-38.8, 5.7], respectively; *P*<0.001) and ΔTLR (-67.6 [-78.1, -56.8] vs. -18.6 [-27.9, 4.0], respectively; *P*<0.001) were reduced in the responder group than those in the non-responder group. According to the results of the receiver operating characteristic curve analysis, ΔTLR showed an excellent predictive value for the MPR of primary HCC lesions (area under curve=0.989, with the optimal diagnostic threshold of -46.15). When using ΔTLR of -21.36% as a threshold, patients with ΔTLR-based metabolic response had superior PFS (log-rank test, *P*=0.001) and OS (log-rank test, *P*=0.016) compared with those without ΔTLR-based metabolic response.

**Conclusion:**

^18^F-FDG PET is a valuable tool for predicting pathological response and prognosis of unresectable HCC patients treated by Lenvatinib combined with PD-1 as a conversion therapy.

## Introduction

Primary liver cancer is the sixth most common cancer and the third leading cause of cancer death worldwide (1). Hepatocellular carcinoma (HCC), as the most common type of primary liver malignancy in the world (75-85% of cases), has shown an increasing prevalence rate globally (2). Although surgical resection is a potentially curative treatment for patients with HCC, the majority of these patients are already in the advanced stage of HCC, and only 40-50% of patients in developed countries with regular physical examination are diagnosed at an early stage (3). Because of liver dysfunction, advanced stage or poor performance, more than half of HCC patients are not candidates of radical resection, resulting in poor prognosis (4, 5).

Non-surgical local or systemic treatment is the predominant choice for most advanced HCC patients (6). In recent years, non-surgical treatment of liver cancer, particularly systemic therapy, has progressed. Especially, for some advanced HCC patients, the original unresectable lesions can be changed to resectable lesions through systemic therapy, which is also called conversion therapy (6). Anti-angiogenic drugs, such as tyrosine kinase inhibitors (TKIs), combined with immunotherapies (e.g., programmed cell death protein 1 [PD-1]) have become an important choice for unresectable or intermediate and advanced HCC, and for conversion therapy of potentially resectable HCC (7). Lenvatinib, a multi-target TKI, was approved for the treatment of unresectable HCC in European countries, USA, Japan, and China (8). Lenvatinib inhibited vascular endothelial growth factor (VEGF) and fibroblast growth factor (FGF) pathways, and suppressed the proliferation signals from VEGF receptor (VEGFR) and FGF receptor (FGFR), which were overexpressed in cancer cells (9, 10). As a type of immunotherapy, the PD-1 blocking monoclonal antibodies act directly on immune cells and block the inhibitory T-cell receptor PD-1, and have also been proven to be effective for the treatment of liver cancer (4). Anti-angiogenic drugs combined with immunotherapy can achieve an objective response rate (ORR) of about 30%, and the median survival for patients receiving this type of therapy can be up to 20 months (11–14). As one of the TKIs combined with immunotherapy, Lenvatinib combined with PD-1 inhibitors have also been confirmed to show a certain therapeutic effect (11, 15–20).

When an unresectable HCC patient successfully receives TKIs combined with immunotherapy and surgery, pathological response is a very important indicator for the postoperative recurrence and long-term survival of the patient (6). Studies have shown that the tumor-free survival of HCC patients after resection is related to pathological response, and the tumor-free survival of patients with pathological response is longer (20, 21). However, how to predict pathological response remains to be investigated. In terms of imaging evaluation, the modified Response Evaluation Criteria in Solid Tumors (mRECIST) criteria were the most common standard to evaluate the therapeutic response of liver lesions (22, 23). However, it is still unclear whether mRECIST can predict pathological response and prognosis of HCC patients after conversion therapy. Although ^18^F-fluorodeoxyglucose positron emission tomography/computed tomography (^18^F-FDG PET/CT) has exhibited a poor sensitivity for the detection of HCC compared with other solid tumors (24), ^18^F-FDG PET/CT has still been used for accurate staging, predicting therapeutic response, and detecting recurrence of HCC (25). In recent years, the metabolic parameters of ^18^F-FDG PET have shown a great value in predicting pathological response and prognosis of various malignant tumors after neoadjuvant therapy (26–28). However, there is no study on the metabolic parameters of ^18^F-FDG PET in predicting pathological response and prognosis of unresectable HCC patients undergoing conversion therapy. The present study aimed to explore the value of ^18^F-FDG PET in predicting pathological response and prognosis of unresectable HCC patients treated with Lenvatinib combined with PD-1 inhibitors as conversion therapy.

## Materials and methods

### Patients

This single-center retrospective study was based on a prospective, single-center, single-arm, investigator-initiated, clinical trial study, which was registered at http://www.chictr.org.cn/(ChiCTR1900023914), and it was approved by the Ethics Committee of the General Hospital of the People’s Liberation Army (Beijing, China). All patients were informed and signed the informed consent form before ^18^F-FDG PET/CT. The study was performed in accordance with the Declaration of Helsinki.

Between July 2019 and March 2023, unresectable HCC patients who underwent pre-treatment and post-treatment ^18^F-FDG PET/CT in the General Hospital of the People’s Liberation Army were retrospectively recruited. The inclusion criteria were as follows:(a) Patients older than 18 years and without a history of other malignance; (b) The diagnosis of HCC was pathologically confirmed by fine-needle biopsy or in accordance with the clinical diagnosis criteria of the American Association for the Study of Liver Diseases (AASLD) (29); (c) Patients who were diagnosed with unresectable HCC, and conversion therapy (combination of Lenvatinib and PD-1 inhibitors) could be performed after clinical evaluation; (d) ^18^F-FDG PET/CT was performed within 2 weeks prior to conversion therapy and within 3 weeks prior to surgery; I No other anti-tumor therapy was given during the treatment using Lenvatinib combined with PD-1 inhibitors, and the drugs were not terminated or changed during the therapy; (f) All patients underwent surgery and had definite postoperative pathological diagnosis; (g) High-quality ^18^F-FDG PET/CT images that could be used for diagnosis.

### PET/CT scanning

All patients underwent ^18^F-FDG PET/CT (Biograph 64; GE Healthcare, New York, NY, USA). Patients were fasted for 6 h with plasma glucose levels under 11.1 mmol/L, and rested for at least 20 min in a quiet waiting room before intravenous administration of ^18^F-FDG (^18^F-FDG was produced by our department, with a radiochemical purity of >95%). Patients were injected with ^18^F-FDG at a dose of 3.70-4.44 MBq/kg (0.10-0.12 mCi/kg). PET/CT scan was performed after 60 min, beginning from the skull base to the upper femur in free-breathing mode. The low-dose CT (LDCT) parameters were as follows: voltage=120 kV, current=100 mAs, rotation=0.8, layer thickness=5 mm, and pitch=1. The parameters of PET included 3-dimensional mode, 2 min/bed (30% overlap), 4-5 beds/person, three iterations, 21 subsets, and Gaussian filter half-height width of 4.0 mm. Images were reconstructed with CT attenuation correction (CTAC) using the ordered subset expectation maximization (OSEM) algorithm.

### Image analysis

Multiparametric analysis prototype (GE Healthcare), a dedicated prototypic post-processing tool, was used for image analysis. Quantitative analyses were performed by two experienced nuclear medicine physicians (WGY and MGY) who were blinded to patients’ clinical data. If there were discrepancies between the two physicians, the process would be repeated two weeks later to reach a consensus. Areas with abnormal uptake of ^18^F-FDG on PET and/or abnormal density on CT were defined as lesions. A two-dimensional region of interest (ROI) was delineated manually according to the boundary of the HCC lesion and portal vein tumor thrombus (PVTT) on each layer of transaxial CT images to form a three-dimensional volume of interest (VOI). Contrast-enhanced magnetic resonance imaging (MRI)/CT was used to accurately determine the VOI. The VOI was applied to the corresponding PET images, which were registered to CT images. To measure a normal liver activity, 3 non-overlapping spherical 1-cm (3)-sized VOIs were drawn in the normal liver on the axial PET images, avoiding the HCC areas on dynamic CT. The SUVmax (maximum standard uptake value) in HCC and PVTT for each patient was calculated by placing a spherical VOI over the sites of the HCC lesions and PVTT. Using the SUVmax of HCC and PVTT and mean SUV of the normal liver, TLR (tumor-to-normal liver standardized uptake value ratio, SUVmax of the tumor/SUVmean of the normal liver parenchyma) and PLR (PVTT-to-normal liver standardized uptake ratio, SUVmax of the PVTT/SUVmean of the normal liver parenchyma) were calculated for each patient. There were no significant differences in terms of SUVmean of the liver parenchyma between the MPR responder group and non-MPR responder group (pre-treatment: 2.41 ± 0.25 vs. 2.60 ± 0.33, *P* = 0.122; post-treatment: 2.38 ± 0.4 vs. 2.31 ± 0.41, *P* = 0.637).

The percentages of post-treatment changes in metabolic parameters were calculated as follows:


$$\text{ΔSUVmax}(\%)=\frac{\text{SUVmax of post-treatment}-\text{SUVmax of pre-treatment }}{\text{SUVmax of pre-treatment }}\times 100\%$$

$$\text{ΔTLR(\%)=}\frac{\text{TLR of pre-treatment - TLR of post-treatment}}{\text{TLR of pre-treatment }}\times 100\%$$

$$\text{ΔPLR(\%)=}\frac{\text{PLR of pre-treatment - PLR of post-treatment}}{\text{PLR of pre-treatment }}\times 100\%$$

Furthermore, ΔSUVmax and ΔTLR of primary HCC lesions, and ΔPLR of PVTT were recorded, respectively.

### Systemic therapy

Conversion therapy mainly included Lenvatinib and PD-1 inhibitors. Patients were treated with intravenous infusion of anti-PD-1 antibodies (dose, 200-240 mg for different drugs), and the vast majority of the data were collected under four treatment regimens (Pembrolizumab 200 mg/q3w, Sintilimab 200 mg/q3w, Toripalimab 240 mg/q3w, and Tislelizumab 200mg/q3w). Lenvatinib was given orally (8 or 12 mg/day, depending on the patient’s weight < 60 or ≥ 60 kg.

### Follow-up during systemic therapy and radiological assessment

All patients were treated regularly and were monitored to assess their response to systemic therapy. Patients’ complete blood count, thyroid, cardiac, liver, renal, adrenal functions, and tumor markers prior to each cycle of PD-1 treatment were assessed. After 3 cycles of treatment with PD-1 inhibitors, tumor response of the patients was evaluated (according to RECIST ver. 1.1 (30) and mRECIST (22) criteria: complete response [CR], partial response [PR], stable disease [SD], and progressive disease [PD], and the resectability of liver cancer was investigated by contrast-enhanced MRI/CT and chest CT. The patients were categorized into responders (CR or PR) and non-responders (SD or PD) according to mRECIST. Immune-related adverse events (irAEs) were assessed using the National Cancer Institute’s Common Terminology Criteria for Adverse Events (ver. 4.0) (31, 32) (**Table S1**).

### Criteria for successful conversion therapy

The criteria for successful conversion therapy were summarized as follows (33): (a) Child-Pugh grade A; (b) Eastern Cooperative Oncology Group Performance Status (ECOG PS) score ≤ 1; (c) Shrinkage or disappearance of metastatic lymph nodes, and the remaining lymph nodes can be removed; (d) No new extrahepatic metastases; I Intact vascular structure (including the inflow and outflow) of the reserved liver; (f) The expected ratio of future liver remnant volume to standard liver volume (FLR/SLV) after resection of tumor-bearing liver is ≥40% in compromised livers and 35% in normal livers. All patients who met the criteria for successful conversion therapy would be informed of the benefits and risks of surgery.

### Histopathological assessment of tumor regression

Surgical specimens were analyzed by two experienced pathologists who were blinded to the patients’ treatment and outcomes. The pathological treatment response (PTR) was classified based on the tumor cellularity. The primary tumors and PPVT were recorded. Major pathological response (MPR, ≤10% residual viable tumor) or complete pathological response (CPR, no residual viable tumor) following immunotherapy was used as endpoints in the great majority of clinical trials (34, 35). Whether patients reached CPR or MPR through HCC lesions and PVTT (if present) was comprehensively considered.

We categorized all patients according to their pathological response into MPR responder and non-MPR responder groups.

### Postoperative therapy and follow-up

Patients continued to receive therapy according to the pathological results and their personal conditions at 4-6 weeks after surgery and clinical evaluation. Serum tumor biomarkers were examined every cycle, and imaging examinations (contrast-enhanced MRI/CT or abdominal ultrasound) were performed every 3 months to monitor HCC recurrence. HCC recurrence was defined as the presence of radiological evidence of new intra- and/or extra-hepatic tumors (36). According to the guidelines, post-recurrence treatments were administered (6). The time of recurrence and death was recorded, respectively.

### Statistical analysis

Quantitative data were expressed as median (interquartile range [IQR]) or mean ± standard deviation (SD). Qualitative data were expressed as number of cases and percentage (n [%]). Homogeneity of variance of the data was verified using Levene’s test, and normal distribution of the data by Shapiro–Wilk test. The student’s *t*-test or Mann-Whitney U test was used to compare ^18^F-FDG PET/CT metabolic parameters among different groups. The categorical variables were analyzed by the Fisher’s exact test or the Chi-square test. The optimal cut-off values for continuous variables were estimated using receiver operating characteristic (ROC) curve analysis with the area under the curve (AUC), and sensitivity, specificity, positive predictive value (PPV), and negative predictive value (NPV) were calculated, respectively. PTR was compared with ^18^F-FDG PET/CT metabolic parameters using Spearman correlation analysis.

The metabolic parameters were dichotomized according to specific cutoff values, which were determined by using ROC curve analysis. Progression-free survival (PFS) was determined as the interval from the start of conversion therapy to the date of disease relapse/progression. Overall survival (OS) was defined as the interval between the conversion therapy and death from any cause. All patients were followed up for at least 6 months (i.e., 2 of 28 patients who was followed up for shorter than 8 months was excluded). Kaplan Meier was used to plot the survival curve and log-rank test of PFS and OS difference was used to evaluate the significance.

The statistical analysis was performed using SPSS 24.0 (IBM, Armonk, NY, USA) and R 4.0.2 (Bell Laboratories, Holmdel, NJ, USA) software. All statistical tests were two-sided and the significance level was set at *P*=0.05.

## Results

### Patients’ characteristics

**Figure 1:**
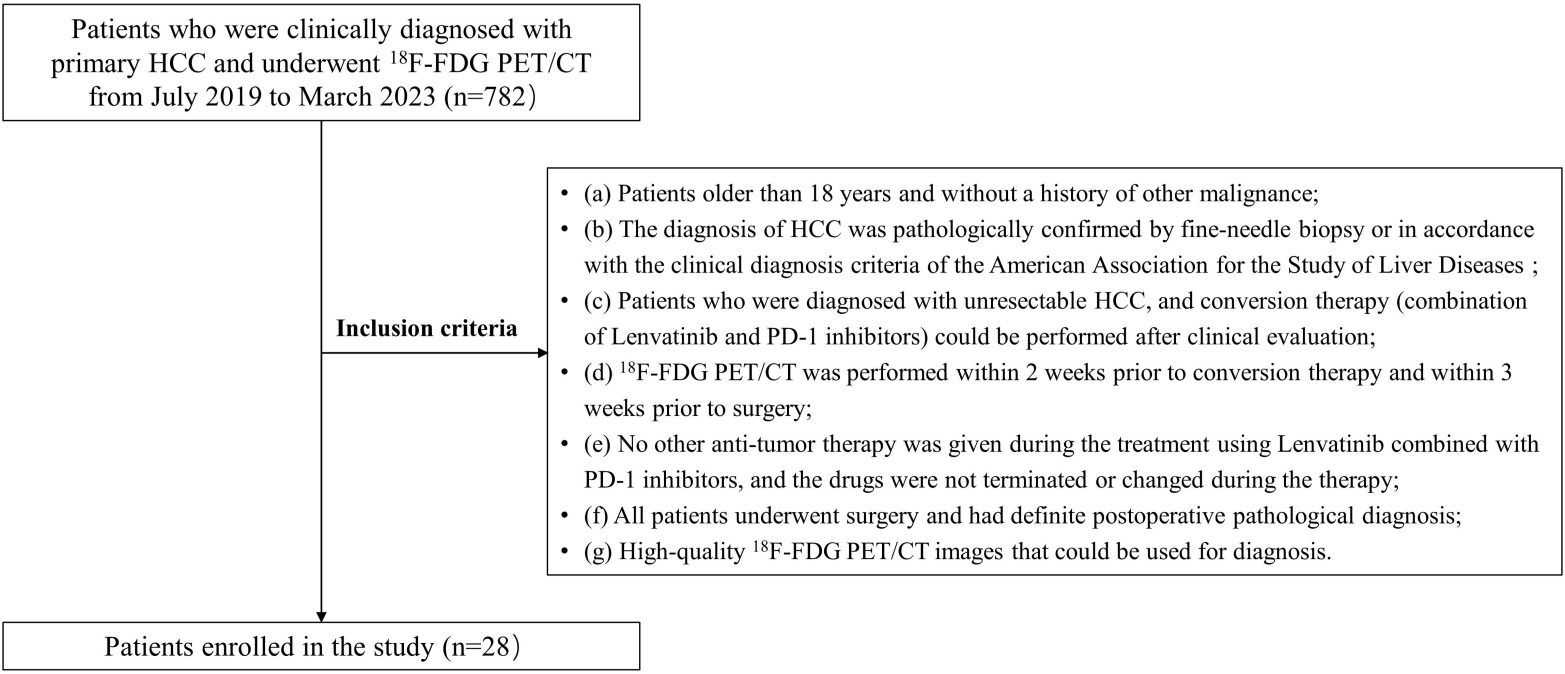
The flow diagram of study. HCC, Hepatocellular carcinoma; ^18^F-FDG PET/CT, ^18^F-fluorodeoxyglucose positron emission tomography/computed tomography.

Eventually, 28 patients underwent surgical excision after successful conversion therapy in our study (24 men; median age: 58.0 years, IQR: 51.8–61.8 years; **Figure 1**). Among them, 11 (11/28, 39.3%) and 17 (17/28, 60.7%) patients were assigned to MPR responder group and non-MPR responder group, respectively. In addition, 5 of 11 patients in the MPR responder group achieved CPR. There was no significant difference in baseline characteristics between MPR responder group and non-MPR responder group in terms of general status (age, gender, body mass index [BMI], alcohol abuse, history of liver diseases, and ECOG PS score), clinical data (Barcelona clinic liver cancer [BCLC] stage, Child-Pugh score, and baseline alpha fetoprotein [AFP] level), imaging findings (tumor diameter, cirrhosis, macroscopic portal vein invasion, extrahepatic metastases), surgical findings (strategy of hepatectomy and R0 resection) and the type of PD-1 inhibitors. The post-treatment AFP level (normal or abnormal), number of tumor and the distribution of mRECIST were significantly different between the two groups (*P*=0.025, *P*=0.025 and *P*=0.001, respectively; **Table 1**). Due to the impact of conversion therapy, only two patients in MPR responder group determined the degree of pathological differentiation, both of whom were poorly differentiated; Among patients in non-MPR responder group, 10 were moderately differentiated, 5 were moderately poorly differentiated, and 1 was poorly differentiated. The median time between the start of conversion therapy and surgery was 107.0 days (IQR: 92.3-133.8 days), the median cycle of conversion therapy was 5.0 (IQR: 4.0-5.8), the median time between the pre-treatment ^18^F-FDG PET/CT and the start of conversion therapy was 4.0 days (IQR: 2.0-7.0 days), the median time between post-treatment ^18^F-FDG PET/CT and surgery was 6 days (IQR: 3.3-8.8 days), and the median time between two ^18^F-FDG PET/CT was 104.5 days (IQR: 90.0-132.3 days). **Supplementary Table 2** shows the details of patients’ conversion therapy and surgery.

**Table 1:** Baseline Clinical and Pathologic Characteristics.

Characteristics	Responder (n=11)	Non-Responder (n=17)	*P*
General status			
**Age**	58.0 (51.0-66.0)	58.0 (48.5-61.0)	0.453^*^
Sex			
** Male**	11 (100%)	13 (77%)	0.132
** Female**	0 (0%)	4 (23%)	
**BMI**	25.0 (22.9-25.9)	23.4 (22.4-25.1)	0.241^*^
**Alcohol abuse**	5 (46%)	9 (53%)	0.699
**History of liver diseases**			0.172
** None**	1 (9%)	3 (17%)	
** Hepatitis B**	10 (91%)	9 (53%)	
** Hepatitis C**	0 (0%)	4 (24%)	
** Other**	0 (0%)	1 (6%)	
**ECOG Performance Status**			1.000
** 0**	11 (100%)	17 (100%)	
** ≥1**	0 (0%)	0 (0%)	
Clinical data			
**BCLC stage**			0.619
** B**	1 (9%)	4 (23.5%)	
** C**	10 (91%)	13 (76.5%)	
**Child-Pugh score**			0.701
** A5**	6 (54.5%)	11 (65%)	
** A6**	5 (45.5%)	6 (35%)	
**Pre-treatment AFP (ng/mL)**			1.000
** <400**	5 (45.5%)	7 (41%)	
** ≥400**	6 (54.5%)	10 (59%)	
**Post-treatment AFP**			0.025
** Normal**	8 (73%)	5 (29%)	
** Abnormal**	3 (27%)	12 (71%)	
**Treatment times (cycle)**	5.0 (5.0-6.0)	4.0 (3.0-5.0)	0.089^*^
Imaging findings			
**Tumor diameter (mm)**	108.0 (73.0-120.0)	86.0 (54.5-113.5)	0.317^*^
**Cirrhosis**	8 (73%)	10 (59%)	0.689
**Macroscopic portal vein invasion**	6 (54.5%)	11 (65%)	0.701
**Extrahepatic metastases**	8 (73%)	6 (35%)	0.053
**Tumor number**			0.025
** Single**	8 (73%)	5 (29%)	
** Multiple**	3 (27%)	12 (71%)	
**mRECIST**			0.001
** CR**	8 (73%)	0 (0%)	
** PR**	2 (18%)	10 (59%)	
** SD**	1 (9%)	6 (35%)	
** PD**	0 (0%)	1 (6%)	
Operation Findings			
**Strategy of hepatectomy**			0.591
** Anatomic resection**	6 (54.5%)	11 (65%)	
** Non-anatomic resection**	5 (45.5%)	6 (35%)	
**R0 resection**	11 (100%)	17 (100%)	1.000
Pathological differentiation			
** Well**	–	0	
** Moderately-Well**	–	0	
** Moderately**	–	11 (65%)	
** Moderately-Poorly**	–	5 (29%)	
** Poorly**	2 (18%)	1 (6%)	
**PD-1 inhibitors**			0.840
** Pembrolizumab**	1 (9%)	1 (6%)	
** Sintilimab**	9 (82%)	14 (82%)	
** Tislelizumab**	0 (0%)	1 (6%)	
** Toripalimab**	1 (9%)	1 (6%)	

*Data are medians with interquartile ranges or numbers of participants with percentages.

^*^Student t test

BMI, Body mass index; BCLC stage, Barcelona Clinic Liver Cancer stage; ECOG PS, Eastern Cooperative Oncology Group performance status; AFP, Alpha fetoprotein; mRECIST, modified Response Evaluation Criteria in Solid Tumors; CR, Complete response; PR, Partial response; SD, Stable disease; PD, Progressive disease.

### Tumor metabolic parameters of ^18^F−FDG PET indicated a significant difference between MPR responder and non−MPR responder groups and predicted pathological response of MPR patients

Pre-treatment ^18^F-FDG PET metabolic parameters were compared between responder group and non-responder group, and there was a significant difference in SUVmax (11.6 [8.7, 16.7] vs. 6.7 [4.5, 10.8], respectively; *P*=0.028) and TLR (5.1 [3.9, 6.5] vs. 2.3 [1.8, 4.0], respectively; *P*=0.022) on pre-treatment scan. The metabolic parameters of post-treatment scan showed no significant difference between MPR responder group and non−MPR responder group (*P*=0.053 and 0.059 for SUVmax and TLR, respectively). ΔSUVmax (%) (-70.0 [-78.8, -48.8] vs. -21.7 [-38.8, 5.7], respectively; *P*<0.001) and ΔTLR (%) (-67.6 [-78.1, -56.8] vs. -18.9 [-27.9, 2.6], respectively; *P*<0.001) were significantly lower in the MPR responder group than those in the non−MPR responder group after conversion therapy (**Table 2**).

**Table 2:** The difference of ^18^F-FDG PET parameters between MPR responders and non-MPR responders in primary lesion.

Parameter	MPR Responders (n=11)	Non-MPR responders (n=17)	*P*
Pre-treatment scan (Scan 1)			
**SUVmax**	11.6 (8.7, 16.7)	6.7 (4.5, 10.8)	0.028^*^
**TLR**	5.1 (3.9, 6.5)	2.3 (1.8, 4.0)	0.022^*^
Post-treatment scan (Scan 2)			
**SUVmax**	3.9 (3.1, 4.2)	5.9 (3.5, 8.7)	0.053^#^
**TLR**	1.7 (1.4, 1.8)	2.6 (1.4, 3.9)	0.059^#^
The percentage changes (Δ%) between pre-treatment scan and post-treatment scan			
**ΔSUVmax (%)**	-70.0 (-78.8, -48.8)	-21.7 (-38.8, 5.7)	<0.001^*^
**ΔTLR (%)**	-67.6 (-78.1, -56.8)	-18.9 (-27.9, 2.6)	<0.001^#^

Data are medians with interquartile ranges in parentheses.

^*^Student t test; ^#^Mann-Whitney test

MPR, Major pathological response; SUVmax, Max standard uptake value; TLR, Tumor-to-normal liver standardized uptake value ratio.

**Figure 2:**
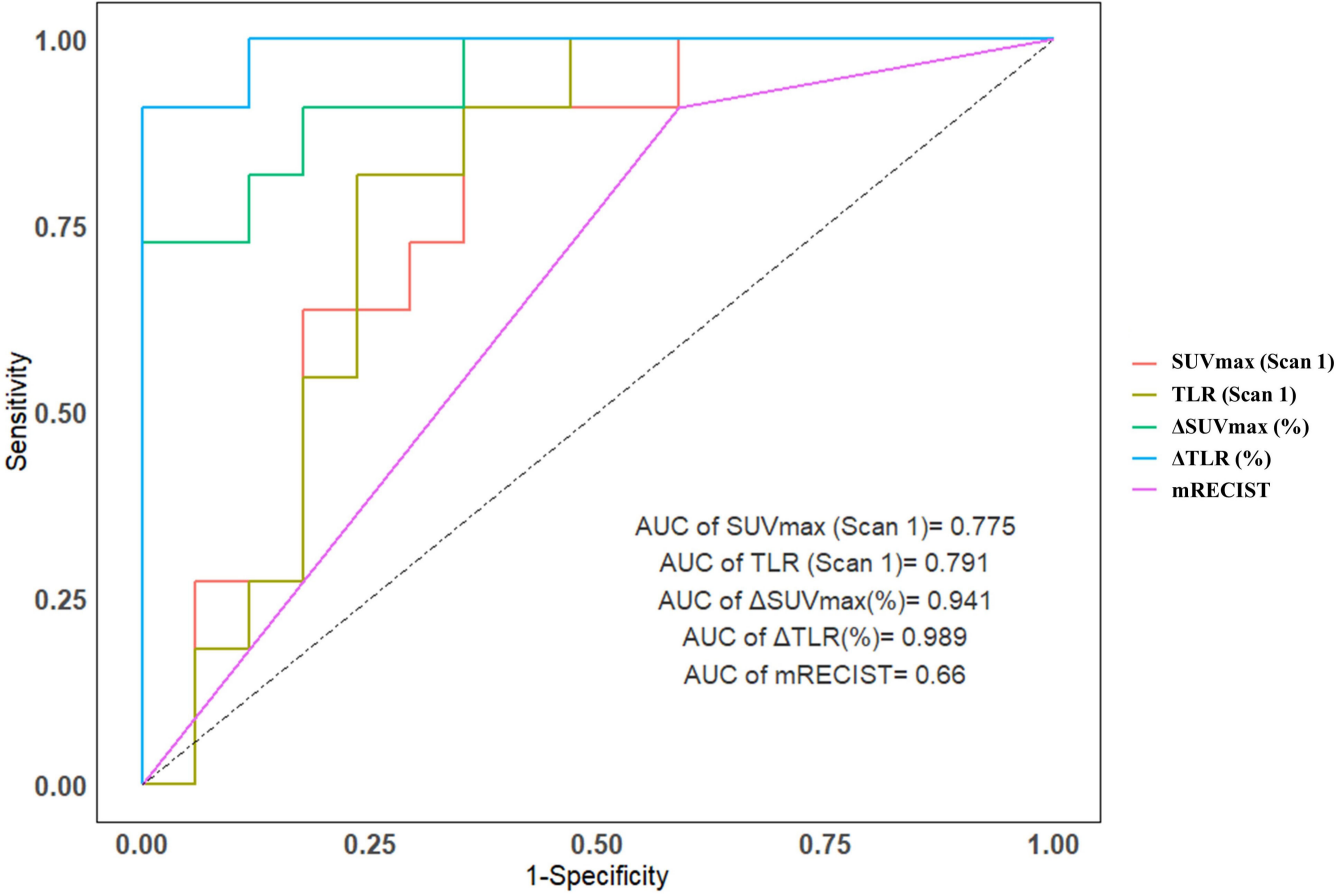
The area under the ROC curve for predicting major pathological response for SUVmax (Scan 1), TLR (Scan 1), ΔTLRand ΔSUVmax was 0.775, 0.791, 0.941 and 0.989, respectively, both are above the mRECIST criteria (0.660).

**Figure 3:**
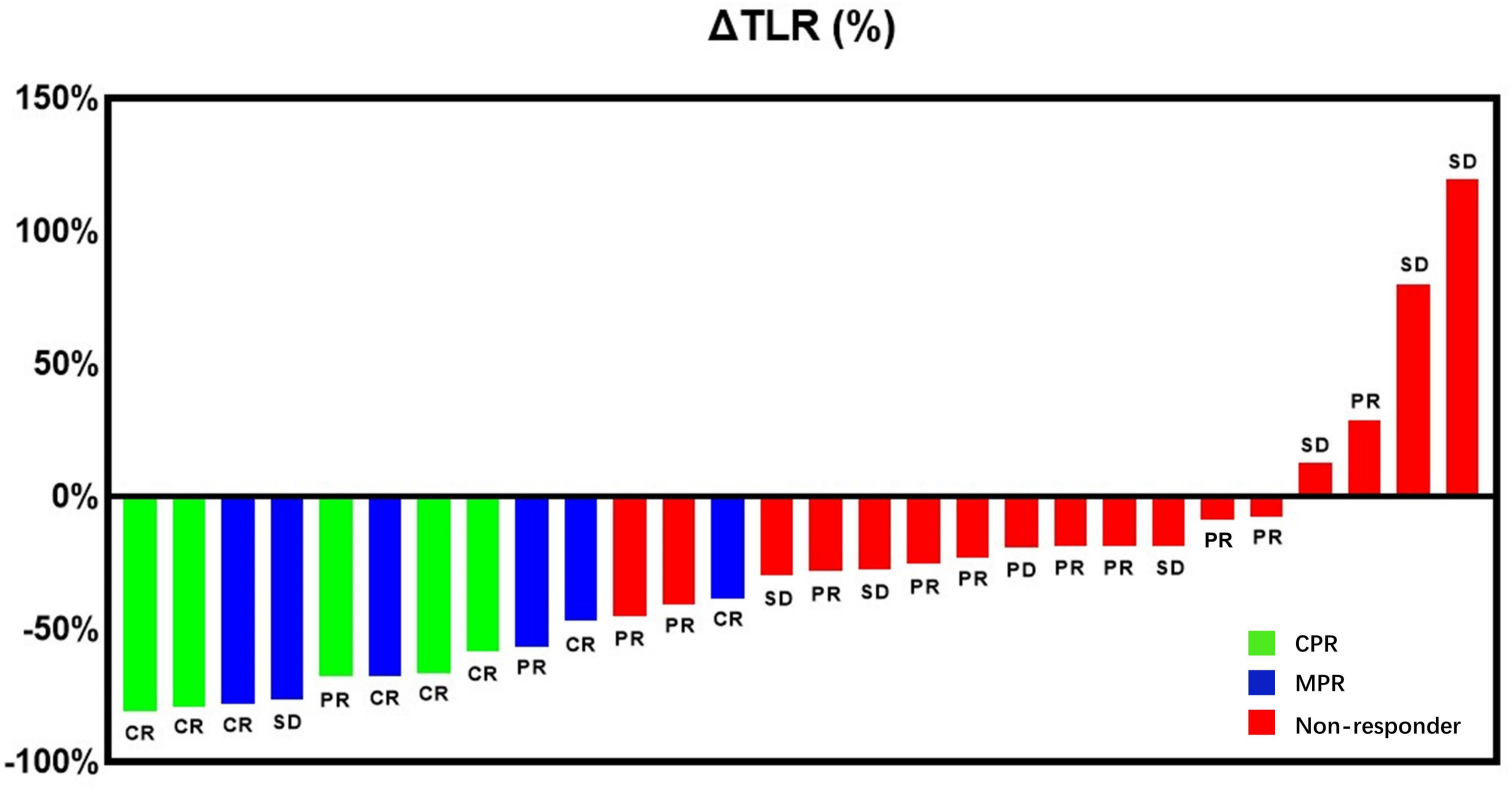
Waterfall plot presenting the percentage of change in primary tumor TLR from baseline to on-treatment per individual patient and the preoperative primary HCC lesions of patients determined by mRECIST criteria. Bar color indicates CPR response (green), MPR response (blue) or non-response (red).

Compared mRECIST and other ^18^F-FDG PET metabolic parameters, ΔTLR (%) showed the largest AUC (AUC=0.989, 95% confidence interval [CI]: 0.962-1.000), with the optimal diagnostic threshold of -46.15. The sensitivity, specificity, PPV, and NPV were 0.909 (0.571-0.995), 1.000 (0.771-1.000), 1.000 (0.655-1.000), and 0.944 (0.706-0.997), respectively (**Table 3** and **Figure 2**). The relationship between the ΔTLR (%) and the mRECIST criteria and pathological response is detailed in **Figure 3**.

**Table 3:** Differential diagnostic efficiency of ^18^F-FDG PET metabolic parameters and mRECIST criteria between MPR-responders and MPR-non-responders.

Parameter	Cut-off	AUC	Sensitivity	Specificity	PPV	NPV
Pre-treatment scan (Scan 1)						
**SUVmax**	8.14	0.775 (0.601-0.950)	0.909 (0.571-0.995)	0.647 (0.386-0.847)	0.625 (0.359-0.738)	0.917 (0.598-0.996)
**TLR**	3.84	0.791 (0.621-0.962)	0.818 (0.478-0.968)	0.765 (0.498-0.922)	0.692 (0.389-0.896)	0.867 (0.584-977)
The percentage changes (Δ%) between pretreatment scan and post-treatment scan						
**ΔSUVmax (%)**	-40.26	0.941 (0.858-1.000)	0.909 (0.571-0.995)	0.824 (0.558-0.953)	0.769 (0.460-0.938)	0.933 (0.660-0.997)
**ΔTLR (%)**	-46.15	0.989 (0.962-1.000)	0.909 (0.571-0.995)	1.000 (0.771-1.000)	1.000 (0.655-1.000)	0.944 (0.706-0.997)
**mRECIST**	–	0.660 (0.457-0.864)	0.909 (0.571-0.995)	0.412 (0.194-0.665)	0.500 (0.279-0.721)	0.875 (0.467-0.993)

mRECIST: CR or PR vs. SD or PD

MPR, Major pathological response; AUC, Area under the curve; PPV, Positive predictive value; NPV, Negative predictive value; TLR, Tumor-to-normal liver standardized uptake value ratio.

### Correlation between ^18^F-FDG PET metabolic parameters and pathological response

**Figure 4:**
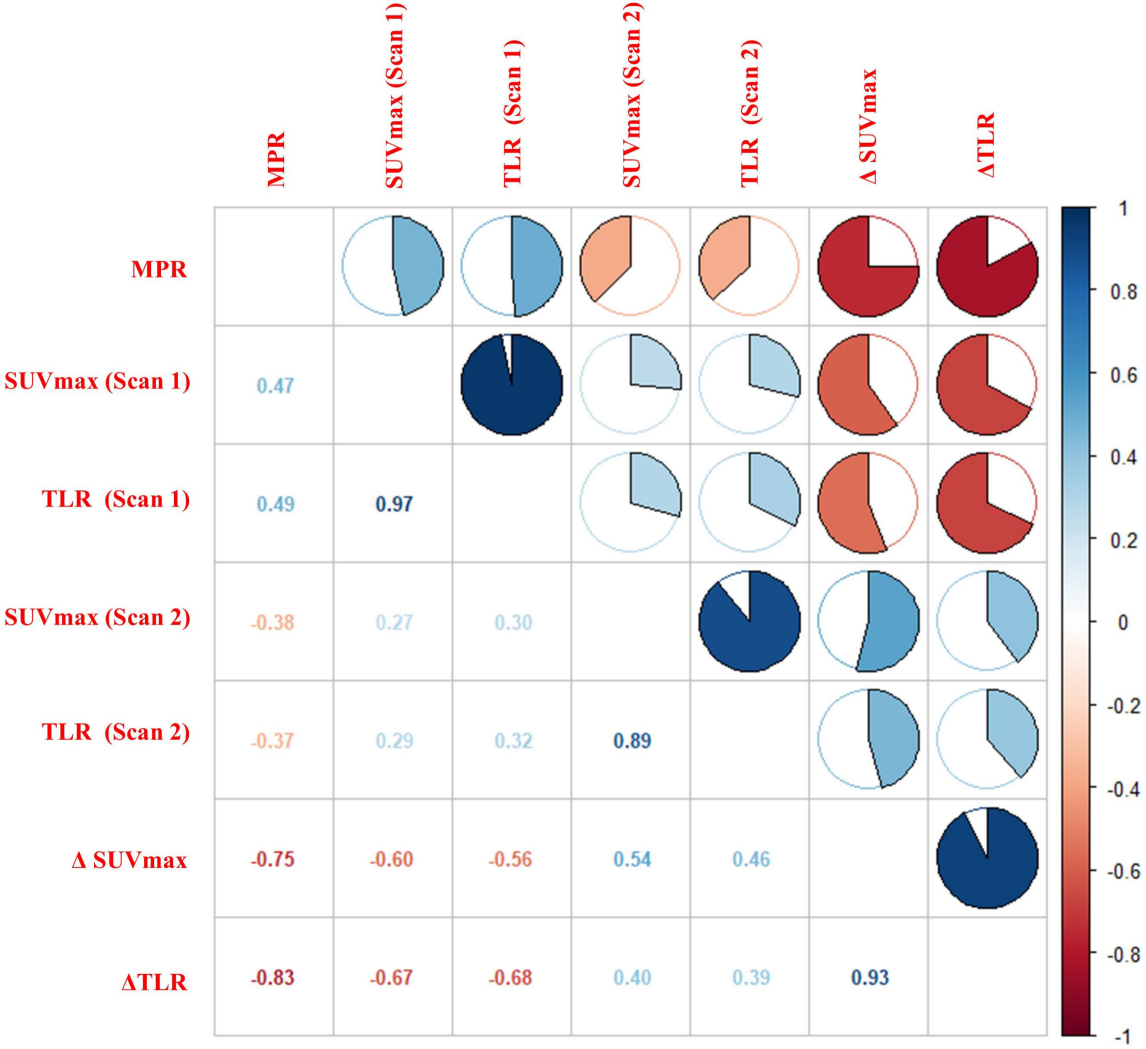
Correlation between ^18^F-FDG PET metabolic parameters and pathological response. The figure showed a strong correlation between ΔTLR and MPR (r = -0.83, *P*< 0.01).

The Spearman correlation analysis was carried out to explore the relationship between ^18^F-FDG PET metabolic parameters and pathological response. The results showed that ΔTLR (%), ΔSUVmax (%), TLR (Scan 1), SUVmax (Scan 1), and SUVmax (Scan 2) were correlated with pathological response, with correlation coefficients (rs) of -0.83, -0.75, 0.49, 0.47, and -0.38, respectively (*P*<0.05). The TLR(Scan 2) showed a lower correlation (r=-0.37), whereas no significant difference was found (*P*>0.05). The results are displayed in **Figure 4**.

### Prognostic value of ^18^F-FDG PET on PFS and OS

**Figure 5:**
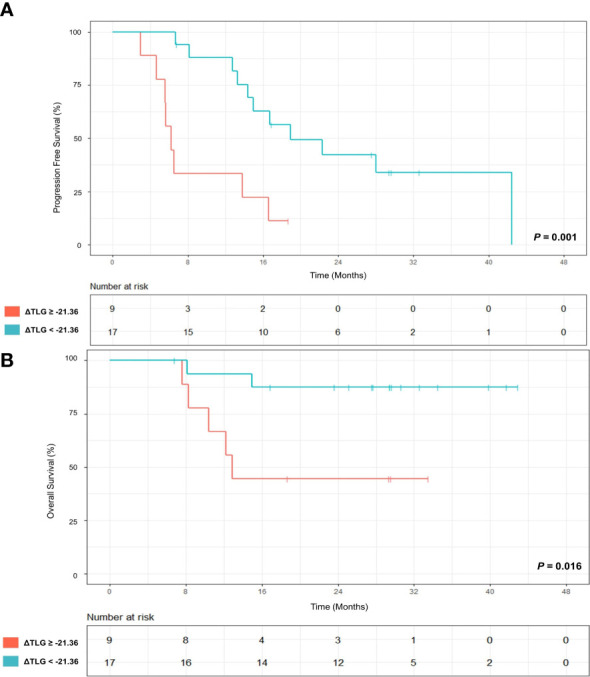
**(A)** Progression-free survival (PFS) of patients with ≥ 21.36% decrease in TLR at primary tumor site (red) from baseline to on-treatment and patients without ≥ 21.36% decrease (green). **(B)** Overall survival (OS) of patients with ≥21.36% decrease in TLR at primary tumor site (red) from baseline to on-treatment and patients without ≥21.36% decrease (green). *P* values were calculated using log-rank test.

The follow-up ended in February 15, 2023. Twopatients waere not included in the analysis due to short follow-up time. During the follow-up period, 19/26 (73.1%) patients showed a disease progression, and median follow-up was 14.7 (IQR:6.6-23.6) months; 7/26 (26.9%) patients died, and median follow-up was 27.6 (IQR: 12.7-31.1) months. When ΔTLR of -46.15% was used as a threshold, patients with ΔTLR-based metabolic response had no superior PFS (log-rank test, *P*=0.112) and OS (log-rank test, *P*=0.218) compared with those without a ΔTLR-based metabolic response. According to ROC analysis, when ΔTLR of -21.36% was used as a threshold, patients with ΔTLR-based metabolic response had superior PFS (log-rank test, *P*=0.001) and OS (log-rank test, *P*=0.016) compared with those without ΔTLR-based metabolic response (**Figure 5**). Patients’ follow-up data are summarized in **Supplementary Table S3**.

### ^18^F-FDG PET identified PVTT involvement

In this study, 17 of 28 (60.7%) patients had macroscopic portal vein invasion. The number of residual tumor cells in PVTT was analyzed in 14 patients. Among them, 9 (9/14, 64.3%) patients were considered as PVTT CPR-responders and 5 (5/14, 35.7%) patients were PVTT CPR-non-responders. The metabolic parameters of pre-treatment scan, post-treatment scan, and the percentage of change in pre-treatment scan and post-treatment scan showed no significant difference between the responder group and the non-responder group (**Table 4**).

**Table 4:** The difference of ^18^F-FDG PET metabolic parameters between CPR-responders and CPR-non-responders in PVTT.

Parameter	CPR-Responders (n=9)	CPR-Non-responders (n=5)	*P*
Pre-treatment scan (Scan 1)			
**SUVmax**	9.2 (5.5, 10.1)	6.5 (5.2, 12.1)	0.970^*^
**PLR**	3.6 (1.9, 3.8)	2.4 (1.9, 4.2)	0.709^*^
Post-treatment scan (Scan 2)			
**SUVmax**	2.2 (2.0, 2.4)	2.7 (2.1, 5.0)	0.147^#^
**PLR**	0.9 (0.8, 1.2)	1.4 (0.9, 2.0)	0.056^*^
The percentage changes (Δ%) between pretreatment scan and post-treatment scan			
**ΔSUVmax (%)**	-76.7 (-79.5, -57.9)	-55.9 (-73.2, -32.6)	0.496^*^
**ΔPLR (%)**	-67.4 (-79.5, -47.1)	-41.9 (-63.6, -23.2)	0.210^*^

Data are medians with interquartile ranges in parentheses.

^*^Student t test; ^#^Mann-Whitney test

CPR, complete pathological response; SUVmax, Max standard uptake value; PLR, PVTT-to-normal liver standardized uptake value ratio.

## Discussion

To our knowledge, this is the first study to report the role of ^18^F-FDG PET in predicting pathological response and prognosis of unresectable HCC patients after treated by Lenvatinib in combination with PD-1 inhibitors as conversion therapy. The results suggested that the differences between the TLR (ΔTLR, %) of pre-treatment ^18^F-FDG PET and post-treatment ^18^F-FDG PET were promising imaging biomarkers for pathological response and prognosis of primary unresectable HCC after treated with the PD-1 blockade in combination with Lenvatinib as conversion therapy. However, ^18^F-FDG PET was not a predictive factor of PVTT pathological response.

CPR has been proven to be an important prognostic factor for patients with multiple malignancies after treatment and surgery, including HCC (37, 38). However, for patients with unresectable HCC, there are few options to achieve CPR, thus, MPR is a good alternative. MPR, defined as equal to 10% residual tumor following neoadjuvant therapy, has also been used as a prognostic factor of malignant tumors, such as non-small cell lung cancer (39), pancreatic cancer (40), and melanoma (41). The possible reason is that patients may not need a complete pathological resolution of the tumor burden to experience clinical benefits, because the main mechanism of the clinical benefits of immunotherapy-based conversion therapy is to initiate an anti-tumor immune response that may systematically seek and destroy microscopic tumor deposits that may lead to tumor recurrence (34). Compared with the traditional RECIST (ver. 1.1) criteria, mRECIST criteria based on CT or MRI were developed to better evaluate the response of liver lesions (6), and they possess some advantages in terms of assessing the degree of pathological response (42). After the treatment takes effect, tumor necrosis appears first, while absorption is relatively slow. Due to the histological and biological changes caused by tumor necrosis, mRECIST criteria are more appropriate for imaging evaluation of conversion therapy (7). However, some studies have shown that mRECIST criteria are only appropriate for assessing pathological response of HCC patients receiving neo-adjuvant therapy before liver transplantation (43). For treatment response, the metabolic parameters of ^18^F-FDG PET also play an important role in predicting HCC (44–46). However, no study has analyzed the pathological response of ^18^F-FDG PET in patients with unresectable HCC after receiving Lenvatinib in combination with PD-1 inhibitors as conversion therapy.

**Figure 6:**
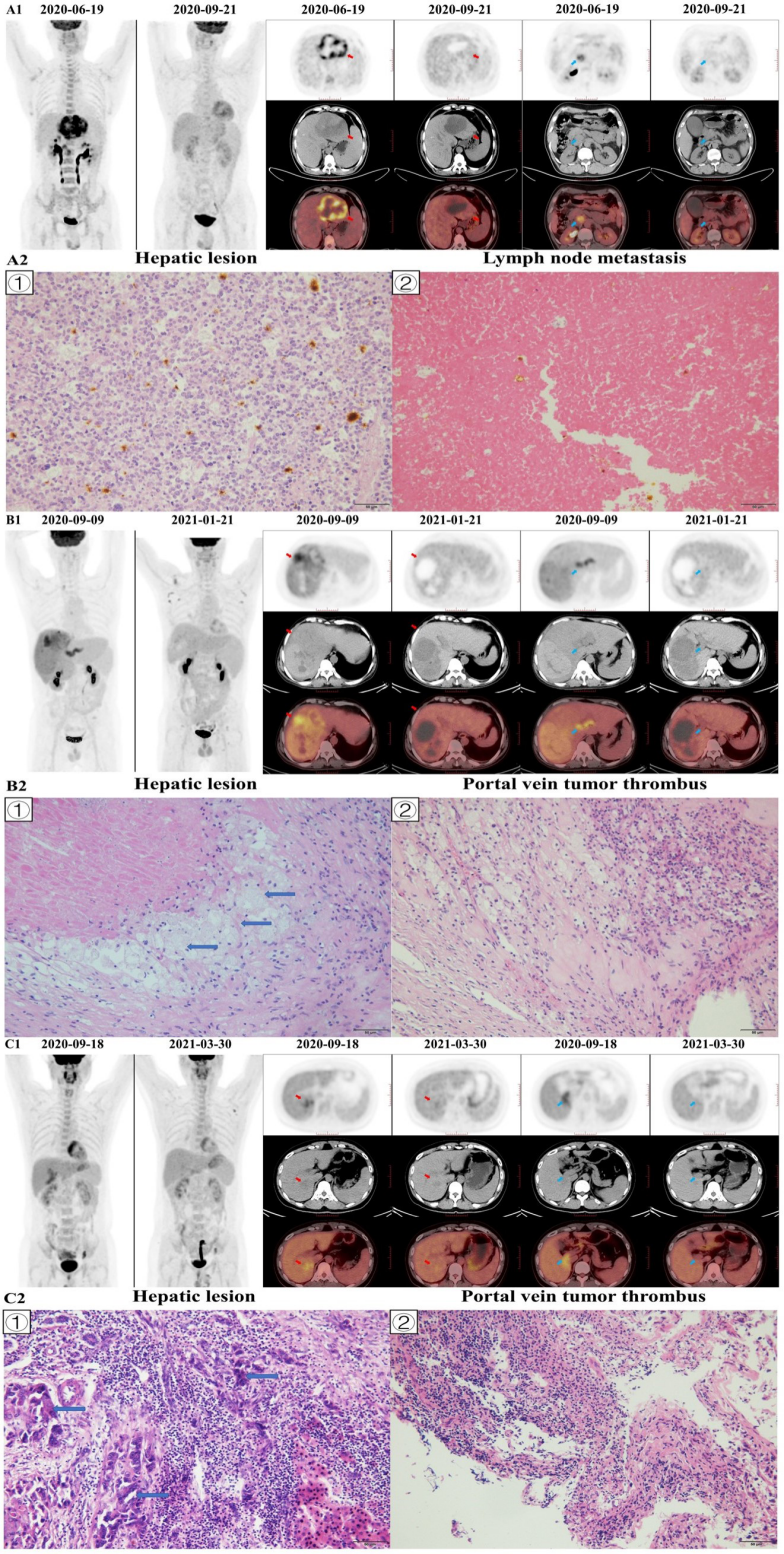
Image A1 shows a 51-year-old man with BCLC-C stage hepatocellular carcinoma in the left hepatic lobe (red arrow), and the patient was accompanied by lymph node metastasis (blue arrow). The hepatic lesion of pre-treatment ^18^F-FDG PET/CT (2020–06–19) showed that tumor-to-normal liver standardized uptake value ratio (TLR) was 8.21, and the hepatic lesion of post-treatment ^18^F-FDG PET/CT (2020–09–21) showed that TLR was 1.80. The percentage of change in TLR was -78.08. The baseline AFP level was 960.4 ng/mL, the baseline tumor diameter was 106 mm, and the Child-Pugh score was 5. The patient had no history of hepatitis and drinking, while had a history of liver cirrhosis. After conversion therapy (4 cycles of Lenvatinib and Sintilimab), the AFP level decreased to 2.95 ng/mL and the tumor diameter decreased to 85 mm. The patient underwent left hemihepatectomy and lymph node dissection, and histopathological evaluation of response revealed major histopathological response to therapy (residual viable tumor cells rate =8%; Image A2 ①), and no residual tumor tissue was found in metastatic lymph nodes; Image A2 ②). The patient died of myocardial infarction 14.9 months later, and there was no recurrence during the follow-up period. Image A2 shows: ① the hepatic tumor, with a small number of tumor cells, some visible mitotic figures, surrounded by a large number of lymphocyte infiltration (×200); ② showed a large number of necrotic tissues in metastatic lymph nodes and cell aggregation (×200). Image B1 shows a 51-year-old man with BCLC-C stage hepatocellular carcinoma in the right hepatic lobe (red arrow), and the patient was accompanied by portal vein tumor thrombus (PVTT; blue arrow). The hepatic lesion of pre-treatment ^18^F-FDG PET/CT (2020–09–09) showed that TLR was 4.22, and the hepatic lesion of post-treatment ^18^F–FDG PET/CT (2021–01–21) showed that TLR was 1.37. The hepatic lesion of pre-treatment ^18^F-FDG PET/CT (2020–09–09) showed that PVTT-to-normal liver standardized uptake value ratio (PLR) was 3.85, and the hepatic lesion of post-treatment ^18^F-FDG PET/CT (2021–01–21) showed that PLR was 0.87. The percentage of change in TLR and PLR was -67.61 and -77.44, respectively. The baseline AFP level was 86.78 ng/mL, the baseline tumor diameter 190 mm, and the Child-Pugh score 6. The patient had no history of hepatitis, while had a history of liver cirrhosis. After conversion therapy (4 cycles of Lenvatinib and Sintilimab), the AFP level decreased to 1.88 ng/mL and the tumor diameter decreased to 127 mm. The patient underwent right hemihepatectomy and PVTT resection. The histopathologic evaluation of primary liver lesion response indicated major histopathological response to therapy (residual viable tumor cells rate<5%; Image B2 ①) and the histopathological evaluation of PVTT response revealed complete histopathological response to therapy (residual viable tumor cells rate=0%; Image B2 ②). No recurrence or death occurred during the follow-up period. Image B2 shows: ① showed the hepatic tumor with no viable tumor cells but foam cells aggregation (blue arrows), and scattered lymphocyte infiltration (×200); ② showed a large area of necrosis in the PVTT, with a large number of inflammatory cell infiltration and foam cell reaction around it, and no obvious viable tumor cells (×200). Image C1 shows a 38-year-old man with BCLC-C stage hepatocellular carcinoma in the right hepatic lobe (red arrow), and the patient was accompanied by portal vein tumor thrombus (blue arrow). The hepatic lesion of pre-treatment ^18^F-FDG PET/CT (2020–09–18) showed that TLR was 1.94, and the hepatic lesion of post-treatment ^18^F-FDG PET/CT (2021–03–30) showed that TLR was 1.39. The hepatic lesion of pre-treatment ^18^F-FDG PET/CT (2020–09–18) showed that PLR was 2.20, and the hepatic lesion of post-treatment ^18^F-FDG PET/CT (2021–03–30) showed that PLR was 1.35. The percentage of change in TLR and PLR was -28.08 and -38.59, respectively. The baseline AFP level was 289.4 ng/mL, the baseline tumor diameter 48 mm, and the Child-Pugh score 5. The patient had no history of hepatitis, while had a history of liver cirrhosis. After conversion therapy (9 cycles of Lenvatinib and Sintilimab), the AFP level decreased to 35.04 ng/mL and the tumor diameter decreased to 23 mm. The patient underwent S7 segmentectomy and PVTT resection. The histopathological evaluation of response revealed no major histopathological response to therapy (residual viable tumor cells rate =85%; Image C2 ①) and the histopathological evaluation of portal vein tumor thrombus response indicated no complete histopathological response to therapy (residual viable tumor cells rate=50%; Image C2 ②). No recurrence or death occurred during the follow-up period. Image C2 shows: ① showed the tumor cell with degeneration, deep staining of the nucleus, obvious atypia (blue arrows), and a large number of lymphocyte infiltration around (×200); ② showed PVTT, visible tumor cells with some cancer tissue degeneration, visible hemorrhage necrosis and foam cell aggregation (×200).

Our previous study indicated that pre-treatment TLR was a potent marker to predict pathological response of HCC patients (BCLC stage C) treated with Lenvatinib and PD-1 inhibitors as conversion therapy (33). In the present study, it was found that pre-treatment TLR could predict MPR (AUC=0.791, sensitivity=81.8%, specificity=76.5%), which is similar to our previous study. One explanation is that the FDG uptake is positively correlated with the content of tumor-infiltrating lymphocytes (TILs), especially T cells (47–49). Besides, the high FDG uptake in HCC may be a valuable predictor of an extremely rapid response to Lenvatinib (50). This may explain the relationship between the high FDG uptake and pathological response, and it is also because more TILs are accumulated in responders’ HCC lesions, and they may more strongly promote the local and systematic enhancement of T cell-mediated anti-tumor immunity by TKIs combined with immunotherapy than non-responders. Therefore, the therapeutic effect of responders was better. This suggested to some extent why there was a greater difference in FDG uptake between pre-treatment and post-treatment ^18^F-FDG PET, and the patient was more likely to achieve MPR. Our results showed that ΔTLR (cut-off value: -46.15%) was the best parameter to predict pathological response of primary HCC lesions, and it was more accurate than mRECIST criteria (**Figure 6**). However, in our patients, four patients showed an increase in ΔTLR. But all four patients were in the non−MPR responder group, and their treatment cycles were relatively short, ranging from 3-5 cycles. All four patients had relapsed, and two died. The reason may be that although the volume of the tumor has decreased, the surviving tumor cells have stronger activity and stronger metabolism compared to before, leading to an increase in FDG uptake, which may lead to postoperative recurrence in these patients. Therefore, using ^18^F-FDG PET to evaluate the conversion therapy effectiveness of unresectable HCC patients at different time points may also help to find a more accurate surgical time. ^18^F-FDG PET may provide more reliable imaging predictors for the timing of operation for unresectable HCC patients treated with Lenvatinib and PD-1 inhibitors as conversion therapy.

As pathological response is associated with prognosis of HCC patients, we hypothesized that FDG metabolic parameters can also predict the prognosis of unresectable HCC patients after receiving Lenvatinib in combination with PD-1 inhibitors as conversion therapy. There are limited data of biomarkers to help decision-making and guide the treatment of advanced HCC (51), and there is no imaging biomarker for prognosis of patients with unresectable HCC after conversion therapy. The present study revealed that ΔTLR (cut-off value: -21.36%) was also an indicator to predict PFS and OS of patients receiving Lenvatinib in combination with PD-1 inhibitors as conversion therapy. Previous studies have shown that the more obvious the reduction of FDG uptake, the better the prognosis (PSF or/and OS) of patients with other malignant tumors treated with TKIs or immunotherapy (52–55). Our present study indicated a potential imaging biomarker of the therapeutic efficacy and prognosis of patients with advanced HCC after treated by conversion therapy.

However, our study found that metabolic parameters of PET could not predict pathological response of PVTT. PVTT plays a major role in the prognosis and clinical staging of HCC (56, 57), some studies have shown that HCC patients with PVTT after neo-adjuvant therapy still have better survival outcomes than those without neo-adjuvant therapy (58, 59). Huang et al. demonstrated that the ORR of Lenvatinib combined with PD-1 inhibitors was 54.5% for macrovascular tumor thrombi (MVTT) and 32.8% for hepatic tumors, and among 17 MVTT patients who achieved ORR, 6 (18.1%) patients underwent surgery (60). Postoperative pathology indicated that 66.7% of patients with PVTT achieved pathological complete necrosis. This confirmed that the conversion therapy of Lenvatinib combined with PD-1 inhibitors had a promising therapeutic effect on PVTT. Therefore, biomarkers are also needed to evaluate pathological response of patients with PVTT. It has been reported that FDG uptake has diagnostic and prognostic value for HCC PVTT (61, 62). However, the components in the tumor thrombus are more complex than the original tumor. After treatment for the tumor thrombus, there may still be many tumor-infiltrating inflammatory cells, which may lead to the increased FDG uptake, disabling metabolic parameters to predict pathological response of patients with PVTT. It is noteworthy that fewer patients were included in this study, and bias was inevitable. More studies are still required to verify our findings.

The present study has some limitations. First, it was a retrospective single-center study and the number of enrolled patients was small. This may bias the study results. However, due to the low proportion of unresectable HCC patients treated with Lenvatinib and PD-1 Inhibitors as a conversion therapy and successfully undergo conversion surgery (63–65), few patients could be included in our study. Second, the follow-up was short, and a longer follow-up period is needed to examine whether ^18^F-FDG PET metabolic parameters on primary tumors can predict survival outcomes of HCC patients after treated with Lenvatinib in combination with PD-1 inhibitors as conversion therapy, followed by surgery. Third, only pathological treatment response of the primary tumor and PVTT was assessed, and there is still a lack of evidence on extrahepatic metastases. Especially, in our study, except for ^18^F-FDG PET, we found that there was a significant difference between MPR responder group and non-MPR responder group in whether the post-treatment AFP levels were normal. This may also provide biomarkers for predicting pathological response, but more research is still needed. Fourth, due to the small number of patients, we were unable to analyze more related factors and predicting biomarker in the survival analysis, such as tumor responses, PVTT, male, baseline AFP level and liver disease history (51, 60, 65, 66). It is therefore essential to comprehensively analyze the related factors in the future large-scale research. Fifthly, since the pathological results of most patients in MPR responder group did not indicate the tumor differentiation, the impact of HCC differentiation on ^18^F-FDG uptake could not be considered. In the future, we will design prospective studies with a longer follow-up and a larger sample size to verify the role of ^18^F-FDG PET in predicting pathological response and prognosis of unresectable HCC patients after treated by Lenvatinib in combination with PD-1 inhibitors as conversion therapy.

## Conclusions

In conclusion, the results of this study suggest that ^18^F-FDG PET is a precious tool for predicting pathological response and prognosis of patients with primary unresectable HCC after treated by Lenvatinib combined with PD-1 inhibitors as conversion therapy. Our study provided valuable markers for clinical decision-making, preoperative evaluation and prognostic prediction of patients with unresectable HCC.

## Data availability statement

The original contributions presented in the study are included in the article/**Supplementary Material**. Further inquiries can be directed to the corresponding authors.

## Ethics statement

The study involving human participants was reviewed and approved by The Ethics Committee of the General Hospital of the People’s Liberation Army (Beijing, China). Written informed consent for participation was not required for this study in accordance with the national legislation and the institutional requirements.

## Author contributions

Conception and design: GYW, WWZ, BXX, SCL, RMW and GYM. Data collation: GYW, WWZ, XHL, ZBW, JJL and JMZ. Statistical Analysis: GYW, RMW and GYM. Article writing: GYW, WWZ, XHL and XDX. Article revision: JJL, JMZ, BXZ, SCL, RMW and GYM. All authors contributed to the article and approved the submitted version.
