# Corrigendum: The role of 18F−FDG PET in predicting the pathological response and prognosis to unresectable HCC patients treated with lenvatinib and PD-1 inhibitors as a conversion therapy

**DOI:** 10.3389/fimmu.2023.1219757

**Published:** 2023-05-22

**Authors:** Guanyun Wang, Wenwen Zhang, Xiaohui Luan, Zhanbo Wang, Jiajin Liu, Xiaodan Xu, Jinming Zhang, Baixuan Xu, Shichun Lu, Ruimin Wang, Guangyu Ma

**Affiliations:** ^1^ Department of Nuclear Medicine, The First Medical Centre, Chinese People’s Liberation Army (PLA) General Hospital, Beijing, China; ^2^ Nuclear Medicine Department, Beijing Friendship Hospital, Capital Medical University, Beijing, China; ^3^ Faculty of Hepato-Pancreato-Biliary Surgery, Chinese People’s Liberation Army (PLA) General Hospital/Institute of Hepatobiliary Surgery of Chinese People’s Liberation Army/Key Laboratory of Digital Hepetobiliary Surgery, People’s Liberation Army, Beijing, China; ^4^ Graduate School, Medical School of Chinese People’s Liberation Army (PLA), Beijing, China; ^5^ Department of Pathology, The First Medical Centre, Chinese People’s Liberation Army (PLA) General Hospital, Beijing, China

**Keywords:** unresectable hepatocellular carcinoma, conversion therapy, major pathological response, prognosis, ^18^F-FDG PET

In the published article, there was an error in the formula in the manuscript.

A correction has been made to **Materials and Methods**, Image analysis, Paragraph 2. This sentence previously stated:

“The percentages of post-treatment changes in metabolic parameters were calculated as follows:


$$\Delta \text{SUVmax}(\%)=\frac{\text{SUVmax of pre-treatment}-\text{SUVmax of post-treatment}}{\text{SUVmax of pretreatment }}\times 100\%$$

$$\text{ΔTLR}(\%)=\frac{\text{TLR of pre-treatment}-\text{TLR of post-treatment}}{\text{TLR of pre-treatment }}\times 100\%$$

$$\text{ΔPLR}(\%)=\frac{\text{PLR of pre-treatment}-\text{PLR of post-treatment}}{\text{PLR of pre-treatment }}\times 100\%”$$

The corrected sentence appears below:

“The percentages of post-treatment changes in metabolic parameters were calculated as follows:


$$\Delta \text{SUVmax}(\%)=\frac{\text{SUVmax of post-treatment}-\text{SUVmax of pre-treatment }}{\text{SUV of pre-treatment }}\times 100\%$$

$$\text{ΔTLR}(\%)=\frac{\text{TLR of post-treatment}-\text{TLR of pre-treatment}}{\text{TLR of pre-treatment }}\times 100\%$$

$$\text{ΔPLR}(\%)=\frac{\text{PLR of post-treatment}-\text{PLR of pre-treatment}}{\text{PLR of pre-treatment }}\times 100\%”$$

The authors apologize for this error and state that this does not change the scientific conclusions of the article in any way. The original article has been updated.

