# Supplementary material for: The role of 18F−FDG PET in predicting the pathological response and prognosis to unresectable HCC patients treated with lenvatinib and PD-1 inhibitors as a conversion therapy
Source: Front Immunol. 2023 May 5;14:1151967. doi: 10.3389/fimmu.2023.1151967 (PMC10196479; doi:10.3389/fimmu.2023.1151967)
Supplement: Supplementary file 1 [file Table_1.docx]

**Supplementary Table 1. The patient’s irAE information**

| Patient No. | irAE during conversion therapy | irAE during Post-operative treatment | Responder/  Non-responder |
| --- | --- | --- | --- |
| 1 | 0 | 0 | Non-responder |
| 2 | 0 | 0 | Non-responder |
| 3 | 0 | Leukopenia | Responder |
| 4 | 0 | 0 | Non-responder |
| 5 | 0 | Skin rash and itch/ Joint pain | Responder |
| 6 | 0 | 0 | Non-responder |
| 7 | Elevated blood pressure/ Bleeding | 0 | Non-responder |
| 8 | 0 | 0 | Responder |
| 9 | Skin rash | 0 | Non-responder |
| 10 | 0 | 0 | Responder |
| 11 | 0 | 0 | Responder |
| 12 | 0 | 0 | Non-responder |
| 13 | Elevated blood pressure/  Liver dysfunction | 0 | Non-responder |
| 14 | Fever/ Skin rash/ Joint pain | 0 | Non-responder |
| 15 | Thrombocytopenia/  Skin rash and itch | 0 | Responder |
| 16 | Liver pain | 0 | Responder |
| 17 | 0 | Skin rash/ Pneumonia | Non-responder |
| 18 | 0 | Abdominal pain/ Oral ulcer/ Bleeding/ Elevated blood pressure/  Skin rash/ Pneumonia | Non-responder |
| 19 | 0 | 0 | Non-responder |
| 20 | 0 | Leukopenia | Non-responder |
| 21 | 0 | 0 | Responder |
| 22 | 0 | Gastrointestinal symptoms | Non-responder |
| 23 | 0 | 0 | Responder |
| 24 | 0 | Skin rash | Non-responder |
| 25 | 0 | 0 | Non-responder |
| 26 | 0 | 0 | Responder |
| 27 | Skin rash | - | Responder |
| 28 | Skin rash/ Gastrointestinal symptoms | - | Non-responder |

irAE: Immune-related adverse events

**Supplementary Table 2. Conversion** **therapeutic regimen for details**

| Patient No. | Therapeutic regimen | Treatment circles | | Days from conversion therapy to surgery | Responder/  Non-responder |
| --- | --- | --- | --- | --- | --- |
| 1 | Lenvatinib + Pembrolizumab | 4 | 94 | | Non-responder |
| 2 | Lenvatinib + Sintilimab | 7 | 138 | | Non-responder |
| 3 | Lenvatinib + Toripalimab | 5 | 109 | | Responder |
| 4 | Lenvatinib + Sintilimab | 5 | 103 | | Non-responder |
| 5 | Lenvatinib + Pembrolizumab | 4 | 105 | | Responder |
| 6 | Lenvatinib + Tislelizumab | 5 | 120 | | Non-responder |
| 7 | Lenvatinib + Sintilimab | 4 | 77 | | Non-responder |
| 8 | Lenvatinib + Sintilimab | 4 | 100 | | Responder |
| 9 | Lenvatinib + Sintilimab | 3 | 84 | | Non-responder |
| 10 | Lenvatinib + Sintilimab | 6 | 139 | | Responder |
| 11 | Lenvatinib + Sintilimab | 7 | 161 | | Responder |
| 12 | Lenvatinib + Sintilimab | 6 | 125 | | Non-responder |
| 13 | Lenvatinib + Sintilimab | 3 | 133 | | Non-responder |
| 14 | Lenvatinib + Sintilimab | 4 | 92 | | Non-responder |
| 15 | Lenvatinib + Sintilimab | 6 | 134 | | Responder |
| 16 | Lenvatinib + Sintilimab | 5 | 103 | | Responder |
| 17 | Lenvatinib + Sintilimab | 3 | 65 | | Non-responder |
| 18 | Lenvatinib + Toripalimab | 3 | 80 | | Non-responder |
| 19 | Lenvatinib + Sintilimab | 3 | 63 | | Non-responder |
| 20 | Lenvatinib + Sintilimab | 9 | 202 | | Non-responder |
| 21 | Lenvatinib + Sintilimab | 14 | 525 | | Responder |
| 22 | Lenvatinib + Sintilimab | 4 | 96 | | Non-responder |
| 23 | Lenvatinib + Sintilimab | 5 | 113 | | Responder |
| 24 | Lenvatinib + Sintilimab | 4 | 89 | | Non-responder |
| 25 | Lenvatinib + Sintilimab | 5 | 93 | | Non-responder |
| 26 | Lenvatinib + Sintilimab | 5 | 115 | | Responder |
| 27 | Lenvatinib + Sintilimab | 5 | 197 | | Responder |
| 28 | Lenvatinib + Sintilimab | 5 | 125 | | Non-responder |

**Supplementary Table 3.**  **The patient’s follow-up information**

| Patient No. | Recurrence | Months from conversion therapy to recurrence | Death | Months from conversion therapy to death | Responder/  Non-responder |
| --- | --- | --- | --- | --- | --- |
| 1 | Recurrence | 6.5 | Death | 12.2 | Non-responder |
| 2 | Recurrence | 5.6 | Death | 8.3 | Non-responder |
| 3 | Recurrence | 28.0 | - | 41.7 | Responder |
| 4 | Recurrence | 4.6 | Death | 12.9 | Non-responder |
| 5 | Recurrence | 6.7 | - | 39.9 | Responder |
| 6 | Recurrence | 5.6 | Death | 7.6 | Non-responder |
| 7 | Recurrence | 6.2 | - | 33.5 | Non-responder |
| 8 | Recurrence | 14.9 | Death | 14.9 | Responder |
| 9 | Recurrence | 14.4 | - | 30.6 | Non-responder |
| 10 | - | 29.6 | - | 29.6 | Responder |
| 11 | Recurrence | 18.9 | - | 29.4 | Responder |
| 12 | - | 32.6 | - | 32.6 | Non-responder |
| 13 | Recurrence | 8.2 | Death | 8.2 | Non-responder |
| 14 | - | 18.7 | - | 18.7 | Non-responder |
| 15 | - | 16.9 | - | 16.9 | Responder |
| 16 | Recurrence | 13.3 | - | 23.6 | Responder |
| 17 | Recurrence | 16.6 | - | 29.5 | Non-responder |
| 18 | Recurrence | 16.7 | - | 25.1 | Non-responder |
| 19 | Recurrence | 13.8 | - | 29.3 | Non-responder |
| 20 | - | 29.4 | - | 29.4 | Non-responder |
| 21 | Recurrence | 22.3 | - | 34.5 | Responder |
| 22 | Recurrence | 3.0 | Death | 10.4 | Non-responder |
| 23 | - | 27.5 | - | 27.5 | Responder |
| 24 | Recurrence | 12.8 | - | 27.7 | Non-responder |
| 25 | Recurrence | 42.5 | - | 42.9 | Non-responder |
| 26 | - | 6.8 | - | 6.8 | Responder |
